# Editorial for the Special Issue on Ultra Precision Technologies for Micromachining, Volume II

**DOI:** 10.3390/mi13111975

**Published:** 2022-11-15

**Authors:** Yukui Cai, Xichun Luo, Jining Sun, Wenlong Chang

**Affiliations:** 1 School of Mechanical Engineering, Shandong University, Jinan 250061, China; 2 Centre for Precision Manufacturing, Department of Design, Manufacturing and Engineering Management, University of Strathclyde, Glasgow G1 1XQ, UK; 3 School of Mechanical Engineering, Dalian University of Technology, Dalian 116024, China

With the increasing demand for ultra-high-precision products and micro-products in fields such as aerospace, national defense, military, transportation, and people’s livelihoods, it has become an important development trend in the field of machining to realize ultra-high-precision machining and miniaturization with a higher level and higher quality. At present, the research on improving the level of mechanical manufacturing mainly focuses on the machining technology and the mechanical equipment itself. Many experts at home and abroad have demonstrated that the improvement in machining technology and mechanical equipment is beneficial to the ultra-precision and miniaturization processing of typical products such as optical free-form surface parts.

In this Special Issue on ultra-precision machining and miniaturization machining, we include seven articles which, respectively, study these machining techniques from different perspectives in order to improve the level and quality of mechanical manufacturing. One of them deals with ultra-precision machining technology, one with an ultra-precision cutting control system, two with miniaturized machining technology, and two with a miniaturized machining system. This Special Issue also includes a review of scanning probe lithography.

In terms of ultra-precision machining technology research, Dongju Chen et al. [1] established an ultra-precision fly cutting surface prediction model under the influence of the properties of KDP crystal materials, and verified the accuracy of the model and determined the frequency range of characteristic signals caused by crystal anisotropy through experiments. In terms of an ultra-precision fast drive cutting system, Fei Ding et al. [2] adopted a deterministic controller design method to preclude the uncertainty associated with controller tuning, which resulted in a control law minimizing positioning errors based on plant and disturbance models. In the aspect of miniaturization processing technology, Zhongwei Chen et al. [3] studied the formation mechanism and control strategy of micro-milling outlet burr, and proposed the best process parameters through experiments. In addition, for abrasive machining, Nikolaos E. Karkalos et al. [4] used the MD model of peripheral nanogrinding using multiple grains with a realistic trajectory to study the effect of nanogrinding at elevated temperatures on grinding forces, chip formation, and subsurface alterations for three different FCC metals (copper, nickel, and aluminum), and determined the grinding efficiency. In terms of miniaturized machining device systems, Zheng Xu et al. [5] proposed a TMSP matching method based on the Kuhn–Munkres algorithm and developed a TMSP automatic matching device with good practicability and repeatability. In order to improve the positioning function of the micro-driven rotation system, Manzhi Yang et al. [6] designed a sub-arc-second micro-drive rotary system consisting of a PZT (piezoelectric actuator) and a micro rotary mechanism, which has a certain reference value in the field of ultra-precision positioning and micromachining. Finally, for scanning probe lithography, Pengfei Fan et al. [7] systematically described the SPL manufacturing mechanism, the latest research progress, technical applications, and characteristics in their review. Meanwhile, the paper also discussed and compared the main SPL nanofabrication approaches.

Through this Special Issue, we hope that more scholars can understand the current research progress on ultra-precision machining and miniaturization machining, and more scholars will be attracted to participate in it.
